# Tumor immunogenicity regulates host immune responses, and conventional dendritic cell type 2 uptakes the majority of tumor antigens in an orthotopic lung cancer model

**DOI:** 10.1007/s00262-024-03828-w

**Published:** 2024-10-03

**Authors:** Ki-Hyun Kim, Seung-jae Kim, Jacob D. Eccles, Christian Ascoli, Gye Young Park

**Affiliations:** 1 Division of Pulmonary, Critical Care, Sleep and Allergy, Department of Medicine, University of Illinois at Chicago, 840 S. Wood St. CSB-920N, M/C719, Chicago, IL 60612 USA; 2 Jesse Brown Veterans Affairs Medical Center, Chicago, IL USA

**Keywords:** Conventional dendritic cell type 2, Lung cancer, Neoantigen, Orthotopic lung cancer model

## Abstract

Human lung cancer carries high genetic alterations, expressing high tumor-specific neoantigens. Although orthotopic murine lung cancer models recapitulate many characteristics of human lung cancers, genetically engineered mouse models have fewer somatic mutations than human lung cancer, resulting in scarce immune cell infiltration and deficient immune responses. The endogenous mouse lung cancer model driven by Kras mutation and Trp53 deletion (KP model) has minimal immune infiltration because of a scarcity of neoantigens. Fine-tuning tumor antigenicity to trigger the appropriate level of antitumor immunity would be key to investigating immune responses against human lung cancer. We engineered the KP model to express antigens of OVA peptides (minOVA) as neoantigens along with ZsGreen, a traceable fluorescent conjugate. The KP model expressing minOVA exhibited stronger immunogenicity with higher immune cell infiltration comprised of CD8^+^ T cells and CD11c^+^ dendritic cells (DCs). Consequently, the KP model expressing minOVA exhibits suppressed tumor growth compared to its origin. We further analyzed tumor-infiltrated DCs. The majority of ZsGreen conjugated with minOVA was observed in the conventional type 2 DCs (cDC2), whereas cDC1 has minimal. These data indicate that tumor immunogenicity regulates host immune responses, and tumor neoantigen is mostly recognized by cDC2 cells, which may play a critical role in initiating antitumor immune responses in an orthotopic murine lung cancer model.

**Supplementary Information:**

The online version contains supplementary material available at 10.1007/s00262-024-03828-w.

## Introduction

Cancer is driven by the accumulation of genetic alterations. Human lung cancer carries the highest mutation burden among all cancer types [1]. Accumulation of genetic alterations and post-translational modifications produces tumor-specific neoantigens [1]. Although some mutations may be neutral, a higher tumor mutational burden (TMB) typically leads to greater tumor immunity [2]. Tumor-specific neoantigens are presented by major histocompatibility molecules and recognized by host immune cells.

Murine lung cancer models are widely used to study cancer immune responses. Orthotopic lung cancer models recapitulate many human lung cancers by reflecting the tumor microenvironment as well as the local immune cell repertoire and immune response within the lung. [3] Genetically engineered mouse models utilize a small number of cancer-driver mutations to promote spontaneous tumor development in orthotopic environments. Notwithstanding, these murine tumors harbor much fewer simple somatic mutations compared to their human counterparts, resulting in less immunogenicity. [4, 5] Kirsten rat sarcoma viral oncogene homolog *(KRAS*) and tumor suppressor p53 (*TP53*) genes are mutated at a high frequency in nonsmall lung cancer (NSCLC) patients. The spontaneous orthotopic lung cancer model expressing mutant *Kras* and deleted *Trp53* has been widely used for lung cancer research. However, this model is characterized by scarcity of CD3^+^ T cells in the tumor and abated immune responses. [5] Contrastingly, stronger tumor immunity may abolish tumor development and progression by triggering robust antitumor immune responses.

It has been challenging to recapitulate the complexity of driver mutations and neoantigens of human cancer in orthotopic lung tumor models. The proper orthotopic lung cancer model with appropriate antitumor immunity is key to investigating immune mechanisms against tumor-specific neoantigens and developing effective immunotherapy. We explored the lung cancer model with orthotopic KP (*Kras* and *Trp53* mutation) lung tumor models expressing variable neoantigens to investigate antitumor host immunity. To overcome the limitations of current murine models of human lung cancer, we generated an orthopedic KP lung cancer model that expresses variable neoantigens and explored its ability to mount antitumor host immunity. This model's ability to mimic the complex interplay between tumor and immune cells holds promise for advancing our understanding of lung cancer immunology and the development of effective immunotherapies.

## Materials and methods

### Mice

C57BL/6, *Scgb1a1-cre*^ERT^, and B6.129-*Kras*^*tm4Tyj*^* Trp53*^*tm1Brn*^/J (LSL-K-ras^G12D^, *p53*^LoxP^, or KP) mice were purchased from Jackson Laboratory (Bar Harbor, ME). *Scgb1a1-cre*^ERT^ mice were crossbred with KP mice to generate *Scgb1a1-cre*^ERT^;KP. All mouse strains were bred in a specific pathogen-free facility maintained by the University of Illinois at Chicago. All mouse experiments were approved by the Institute Animal Care and Use Committees (IACUC) of the University of Illinois at Chicago and Jesse Brown VA Medical Center. Mouse genotypes from tail biopsies were determined using real-time PCR with specific probes designed for each gene (Transnetyx, Cordova, TN). Age-matched male mice 6 to 12 weeks old were used for the experiments.

Survival analysis was calculated from the day of cancer cell engagement until the humane endpoints were reached. Mice euthanized due to reaching the humane endpoint of 20% body weight loss were recorded as events, while those surviving until the study endpoint without reaching the humane endpoint were censored. This study was conducted in strict accordance with the recommendations in the Guide for the Care and Use of Laboratory Animals of the Nation Institutes of Health.

### Tumor induction

To induce mouse lung adenocarcinoma, a suspension of tamoxifen (MP Biomedicals) in corn oil (Sigma-Aldrich) was administered via intraperitoneal injection (100 mg/kg) to eight-week-old *Scgb1a1-cre*^ERT^; KP mice every three days for two weeks. Tumor-bearing *Scgb1a1-cre*^ERT^; KP mice were 13 weeks old at the time of sacrifice for cancer cell isolation.

### Cell sorting and culture

Lung tumors were dissociated into single-cell suspension through perfusion and enzymatic digestion as described previously (Methods Mol Bio. 2018, 1799, 59-69). FACS sorting was performed using the MoFlo Astrios cell sorter at the UIC RRC Flow Cytometry Core. The epithelial cells were sorted based on their expression profile, specifically negative for CD45-APC-Cy7 (BioLegend, 30-F11), CD31-PE (BioLegend, 390), and CD140a-PE-Cy7 (BioLegend, APA5) antibodies and positive for EpCam-APC (BioLegend, G8.8) antibody. Cells were collected into a solution of 20% FBS (fetal bovine serum) in RPMI1640 medium and subsequently cultured in media containing 10% FBS in RPMI1640.

### FACS analysis

Whole lung cells were prepared as described previously [6]. APC conjugated antibodies against mouse CD3 (17A2), B220 (RA3-6B2), NK-1.1(PK136), TER-119 (TER-119), CD64 (X54-5/7.1) and F4/80 (BM8) were used for linage exclusion. Antibodies against mouse CD45-APC-Cy7(30-F11), CD11c-PE-Cy7(N418), I-A/I-E PE/Dazzle™ 594 (M5/114.15.2), CD172a-AF700 (P84) and XCR1-BV510 (ZET) were used for cDC1 and cDC2 staining. All flow cytometry antibodies were purchased from BioLegend. Samples were run through Gallios flow cytometer and analyzed by Kaluza software (Beckman Coulter, Pasadena, CA).

### Viral vector generation and transduction

In order to express the stable activity of *Cre* recombinase consistently, cancer cells were transduced with Cre-expressing lentivirus (Gentarget, LVP412), and a GFP-Puromycin fusion dual marker was utilized to select the positively transduced stable cells via GFP sorting and antibiotic selection.

ZsGreen-fused minOVA construct was generously provided by Dr. Stefani Spranger (M.I.T.) [7] and cloned into the pBABE lentiviral expression vector. Subsequently, pBABE-ZsGreen-minOVA was co-transfected into 293T cells along with pCMV delta R8.2 and pCMV-VSVg vectors to generate the lentiviral vector (UIC Viral Core). KPLCLS cells were transduced with the ZsGreen-minOVA expressing lentivirus, and bleomycin was used to select the positively transduced stable cells.

### MNU (*N*-methyl-*N*-nitrosourea) treatment

Cancer cells were cultured until they reached approximately 70% confluency, followed by exposure to 100 ug/ml of MNU (Chem Service, NG-17031) dissolved in PBS for 45 min. Post MNU treatment, the cells underwent PBS washes before being replenished with fresh culture media. Ahead of each subsequent MNU exposure, the cells were passaged a minimum of three times, continuing this cycle for up to seven times. 

### Orthotopic transplantation of *cancer* cells via intratracheal delivery

The cancer cells were resuspended in 50 μl of serum and antibiotic-free RPMI, supplemented with 0.01 M ethylenediaminetetraacetic acid (EDTA). For intratracheal administration of cancer cells, we used the previously published method with minor modifications [8]. The mice were anesthetized by intraperitoneal injection of ketamine (100 mg/kg) and xylazine (10 mg/kg) mixture. Anesthesia depth was monitored by checking for lack of response to toe pinch. The anesthetized mouse was securely positioned on an intubation platform, with its back against the platform and its front teeth gently secured to the platform edge. The mouse’s mouth was opened, and the tongue was gently pulled out to expose the trachea. A catheter was carefully inserted into the trachea, ensuring that the tip reached the level of the front teeth. The 50 μl volume of cancer cell suspension was slowly dispensed through the catheter into the trachea, allowing the mouse to inhale the entire volume. Once the entire volume was inhaled, the catheter was gently removed from the trachea. At the desired post-administration time, mice were euthanized, and lungs or lymph nodes were collected.

### Histology and immunohistochemistry (Halo quantitation)

Tissues were formalin-fixed, paraffin-embedded, and 5-µm sections prepared. To quantify tumor burden, lung tissue sections were stained with H&E. The proportion of involved cancer area was determined by measuring the total cancer area/total area of tissue using the HALO platform (Indica Labs), an AI-based tissue classifier module. HALO platform quantification was performed by the UIC Pathology Core in an unbiased, investigator-blinded manner.

For immunohistochemistry, epitopes were unmasked by incubation with sodium citrate pH 6.0 and incubated with primary antibodies for CD45 (Cell signaling, 70,257), CD8a (Abcam, ab217344), CD11c (Cell signaling, 97,585), and GFP (Santa Cruz, sc-9996) overnight. Secondary antibodies were conjugated with horseradish peroxidase (Vector laboratories, MP-7401), or alkaline phosphatase (Vector laboratories, MP-5402), and brown 3,3’-diaminobenzidine (DAB) kit for horseradish peroxidase (Vector laboratories, SK-4105), or red substrate kit for alkaline phosphatase (Vector laboratories, SK-5100) were used as chromogen, respectively. Samples were counterstained with hematoxylin.

### Statistics

Data are expressed as mean ± S.E.M. Tukey’s one-way ANOVA was performed to determine the probability of statistically significant differences (*p* values) and recorded in figure legends. Survival data, including time to event (humane endpoint), were analyzed using the Kaplan–Meier method (*n* = 4–5 mice/group). Statistical significance for survival between populations was analyzed by a log-rank test and adjustment for multiple comparisons was performed utilizing the Bonferroni-Dunn method. All statistics were performed on GraphPad Prism 10.2.3.

## Results

### Generation of orthotopic KP lung tumor model expressing neoantigens with tumor traceable marker

**Fig. 1:**
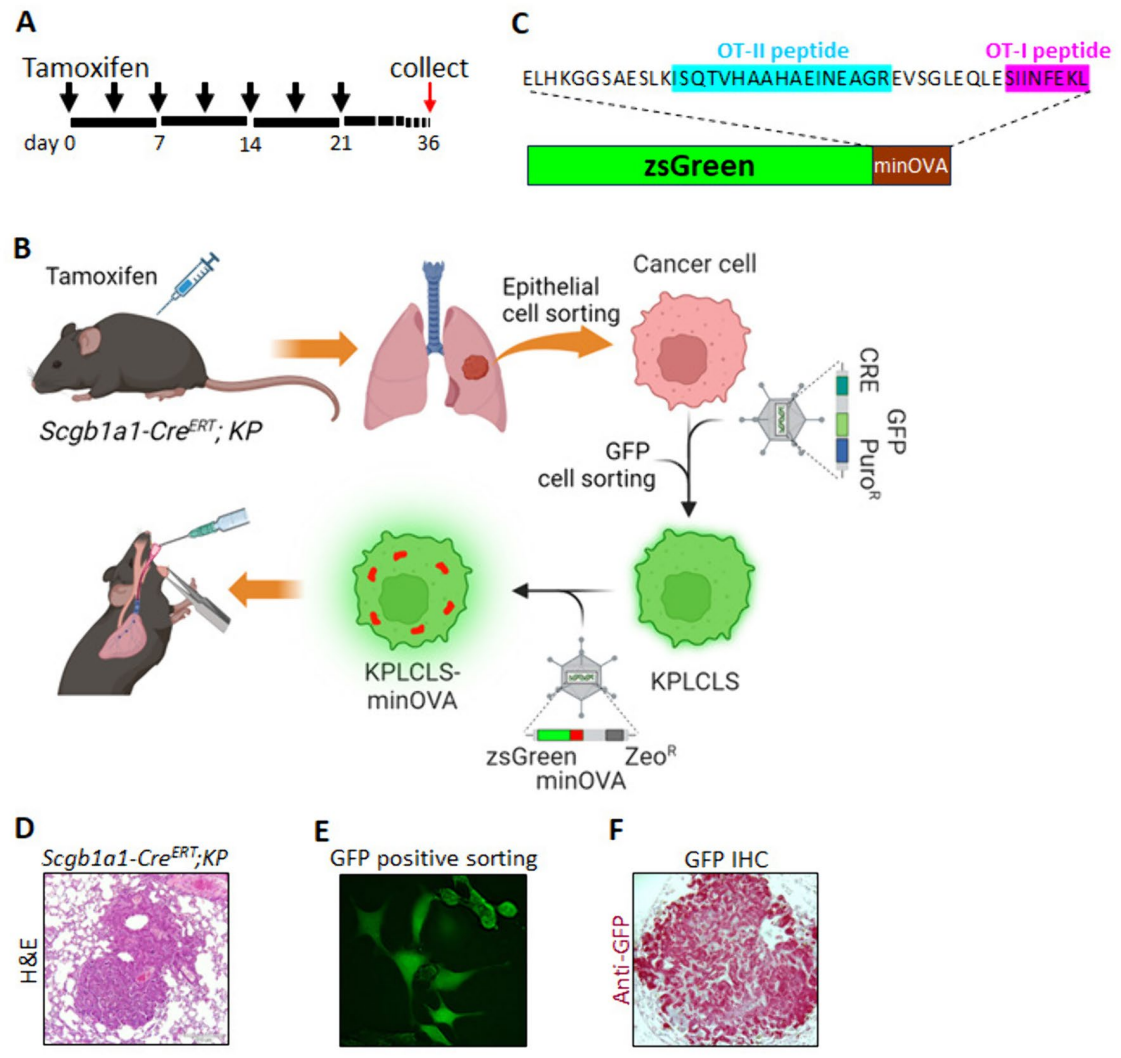
**Generation of mouse lung adenocarcinoma cells.** **A** Schematic representation of the tamoxifen intraperitoneal injection schedule. Black arrowheads denote the time points at which tamoxifen was administered to each mouse. Lung tissues were harvested on day 36 for isolation of cancer cells. **B** Experimental workflow illustrating the overall process. *Scgb1a1-Cre*^*ERT*^; KP mice were injected with tamoxifen according to the schedule described above, followed by lung perfusion with digestion enzymes. Epithelial cells were isolated via fluorescence-activated cell sorting (FACS) and cultured. Isolated cells were transduced with a lentivirus bearing Cre to establish a stable cell line. Subsequently, a lentivirus carrying ZsGreen fused with minOVA was transduced into KPLCLS cells. Cancer cells were then engrafted intratracheally. **C** Amino acid sequence information of ZsGreen fused with minOVA. **D** Hematoxylin and eosin (H&E) staining of lung cancer induced by tamoxifen injections. **E** GFP + cells were sorted by FACS and cultured in media supplemented with Puromycin. **F** KPLCLS-minOVA cells were inoculated via the intratracheal route and the lung tissues were collected after 36 days. Representative histological sections of lung cancer were stained with anti-GFP antibody (red)

*Kras*^*LSL−G12D*^; *Trp53*^*flox/flox*^ (hereafter KP mice) express endogenous mutant *Kras* and deleted *Trp53* alleles (Jackson lab. #032435). The KP mice were crossbred with airway-epithelial cell *Cre*-expressing mice (*Scgb1a1-cre*^ERT^, Jackson lab. #016225) to generate a conditional lung adenocarcinoma model (*Scgb1a*1-*Cre*^ERT^; *KP* mice). Upon tamoxifen injection, the mice developed lung adenocarcinoma, similar to pathologic human lung cancer (Fig. 1D). On day 36, the tumor cells were isolated from the lung as described in methods (Fig. 1A & B). The isolated KP cancer cells were transduced with the lentiviral vector expressing *Cre*-nuclease and selection markers (Fig. 1E). The purified KP lung cancer cells (KPLCLS cells) were further transformed by transduction of the lentiviral vector containing minOVA fused with ZsGreen fluorescent, resulting in generating KPLCLS-minOVA cells (Fig. 1B). MinOVA serves as a neoantigen, and ZsGreen allows us to track the tumor cells. MinOVA comprises two peptides of OT-I (SIIINFEKL) and OT-II (ISQAVHAAHAEINEAGR) (Fig. 1C), which are much less immunogenic compared to OVA whole protein, are still capable of triggering host immune responses [7, 9, and 10]. ZsGreen, an improved version of green fluorescent protein (GFP), is much brighter and more photostable than GFP. KPLCLS-minOVA cells were subsequently introduced via the intratracheal route into mice with an immunologically identical background of the *Scgb1a1*-*Cre*^ERT^; *KP* mice (Fig. 1B). We confirmed successful tumor engraftment in the peribronchial area with GFP-positivity in the tumor cells. The pathology showed a typical lung adenocarcinoma pattern (Fig. 1F).

### Loaded tumor cell count relates to *cancer* burden and determines survival

**Fig. 2:**
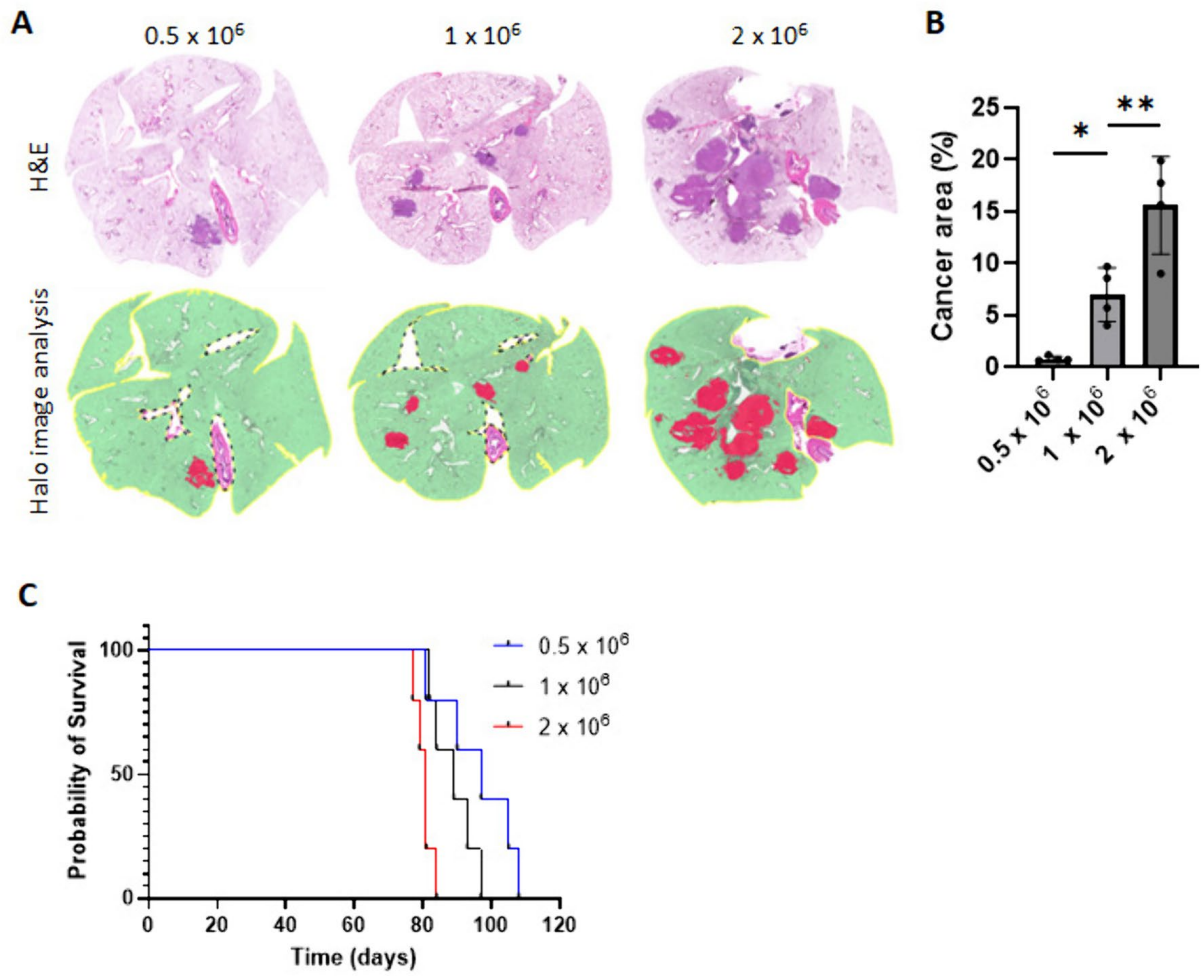
**Impact of varied doses of KPLCLS-minOVA cells on tumor burden and survival in mice.** **A** Three different doses (0.5, 1.0, and 2.0 × 10^6^) of KPLCLS-minOVA cells were inoculated via the intratracheal route, and the lung tissue was collected after 28 days. Formalin-fixed mouse lung tissues were H&E stained and **B** quantified by the AI-powered HALO software. *n *= 4 each. *P < 0.05, **P < 0.01, Tukey’s one-way ANOVA. **C** Survival was monitored and found to be significantly reduced with increasing dose of KPLCLS-minOVA cells. *n* = 5 each. Log-rank (Mantel-Cox) test, *P* value: 0.0034. Post-hoc analysis (Bonferroni-Dunn method) demonstrated that this difference was driven by a dose of 2.0 × 10^6^ (adjusted *P* values: 0.5163, 0.0336, and 0.0462 for 0.5 vs 1.0, 0.5 vs 2.0, and 1.0 vs 2.0; respectively)

Next, we tested the effect of loaded cell count on tumor burden and mouse survival. KPLCLS-minOVA cells were inoculated into mice with identical immunologic backgrounds via the intratracheal route at three different doses, 0.5, 1.0, and 2.0 × 10^6^ (Fig. 2A). A similar level of cross-sectional cuts was performed on day 28 after inoculation to compare dose-dependent tumor characteristics (Fig. 2A). Lung cross-sections at all doses shown classical adenocarcinoma in the peribronchial area and unbiased measurement of tumor-involved area, as quantified by the AI-powered HALO software using entire lung fields, demonstrated that greater inoculated tumor cell numbers are associated with increased tumor-involved area (Fig. 2B). Accordingly, mouse survival was found to be inversely proportional to the dose of inoculated tumor cells (Fig. 2C).

### Stronger tumor immunogenicity suppresses tumor growth

**Fig. 3:**
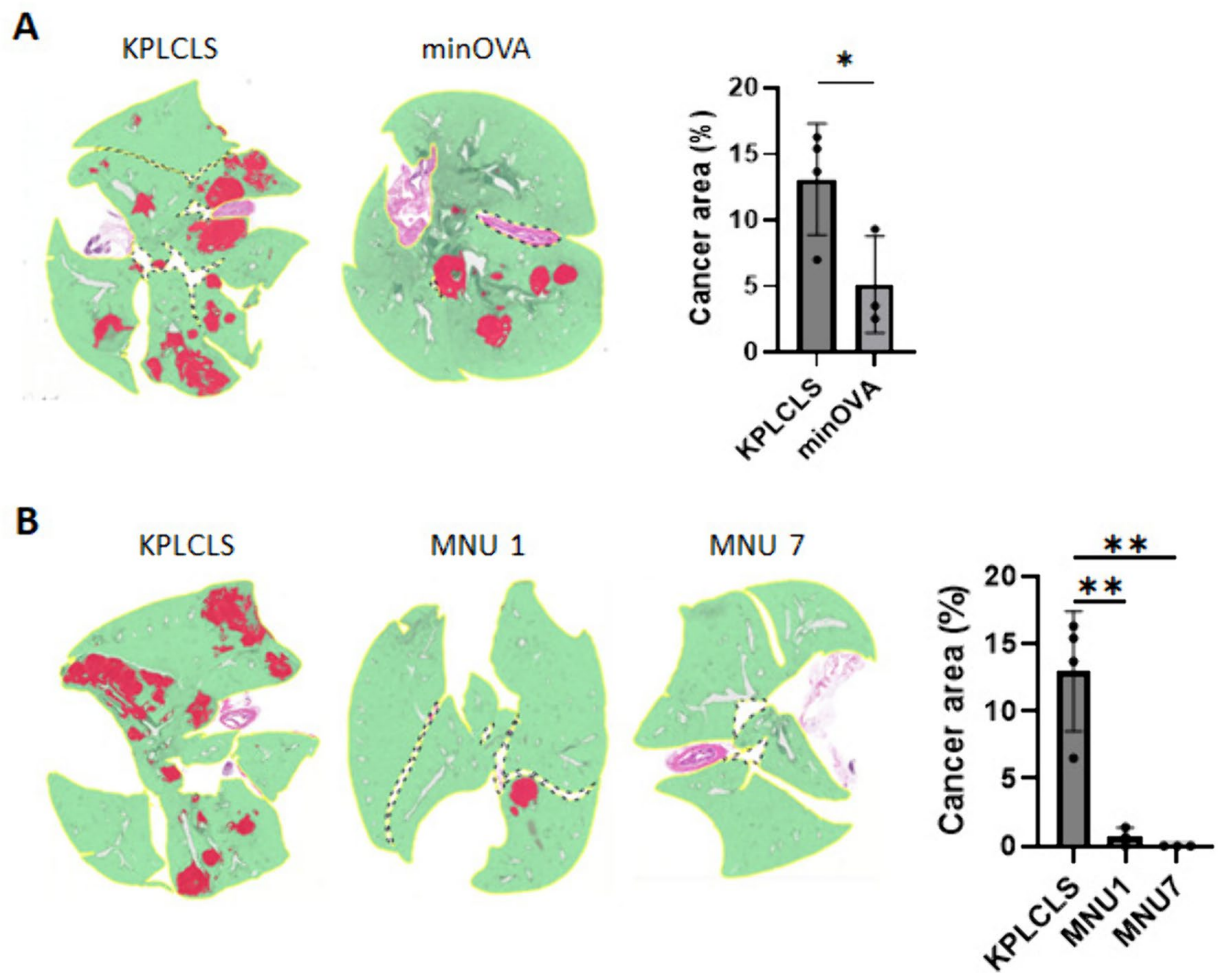
**Tumor immunogenicity suppresses tumor growth.** **A** KPLCLS or KPLCLS-minOVA cells, and **B** KPLCLS or MNU treated KPLCLS cells (1.0 × 10^6^) were intratracheally injected into WT mice, and the lung tissues were collected after 28 days. Formalin-fixed tissues were stained with H&E and the tumor areas were quantified using AI-powered HALO software. (*n* = 4 for KPLCLS, n = 3 for KPLCLS-minOVA). *P < 0.05, **P < 0.01, Tukey’s one-way ANOVA

The KPLCLS-minOVA cells were derived from the KPLCLS by adding the minOVA peptides, as shown in Fig. 1B. Thus, the KPLCLS-minOVA cells have stronger immunogenicity than their origin. We compared tumor growth between these two tumor cells and found that the KPLCLS-minOVA cells had suppressed tumor growth (Fig. 3A). We further examined the correlation between immunogenicity and tumor growth using a carcinogen exposure model. Carcinogen exposure increases tumor mutational burden (TMB), enhancing neoantigen load and tumor immunogenicity. [11] The KPLCLS cells were exposed to 100 ug/mL of MNU. (*N-*methyl-*N*-nitrosourea), as previously reported [12]. The cells were exposed repeatedly for up to seven cycles. After the exposure, the cell growth rate remained the same as that of the unexposed (data not shown), as shown in the other group [12]. An equal number of two MNU-treated cells and their parental KPLCLS-minOVA cells were inoculated into immune-deficient NSG mice ( NOD *scid* gamma: NOD.Cg-*Prkdc*^*scid*^* II2rg*^*tm1wjl*^*/SzJ*, Jackson Lab#005557) and compared their tumor growth. Their growth rates were similar, although MNU7 (seven-cycle treated cells) showed a slightly reduced growth rate (Supplementary Fig. 1). Next, in the same way, an equal number of the cells were inoculated into immunocompetent wild-type mice with the same background. The KPLCLS exposed to one cycle of MNU (MNU1) resulted in a marked reduction in the number of tumor nodules and involved areas, and the cells exposed to seven cycles of MNU (MNU7) failed to implant in the lung despite growing well in vitro culture and the immune-deficient NSG mice (Fig. 3B).

### KPLCLS-minOVA cells promote greater infiltration of immune cells, and cDC2, not cDC1, uptakes most *cancer* particles

**Fig. 4:**
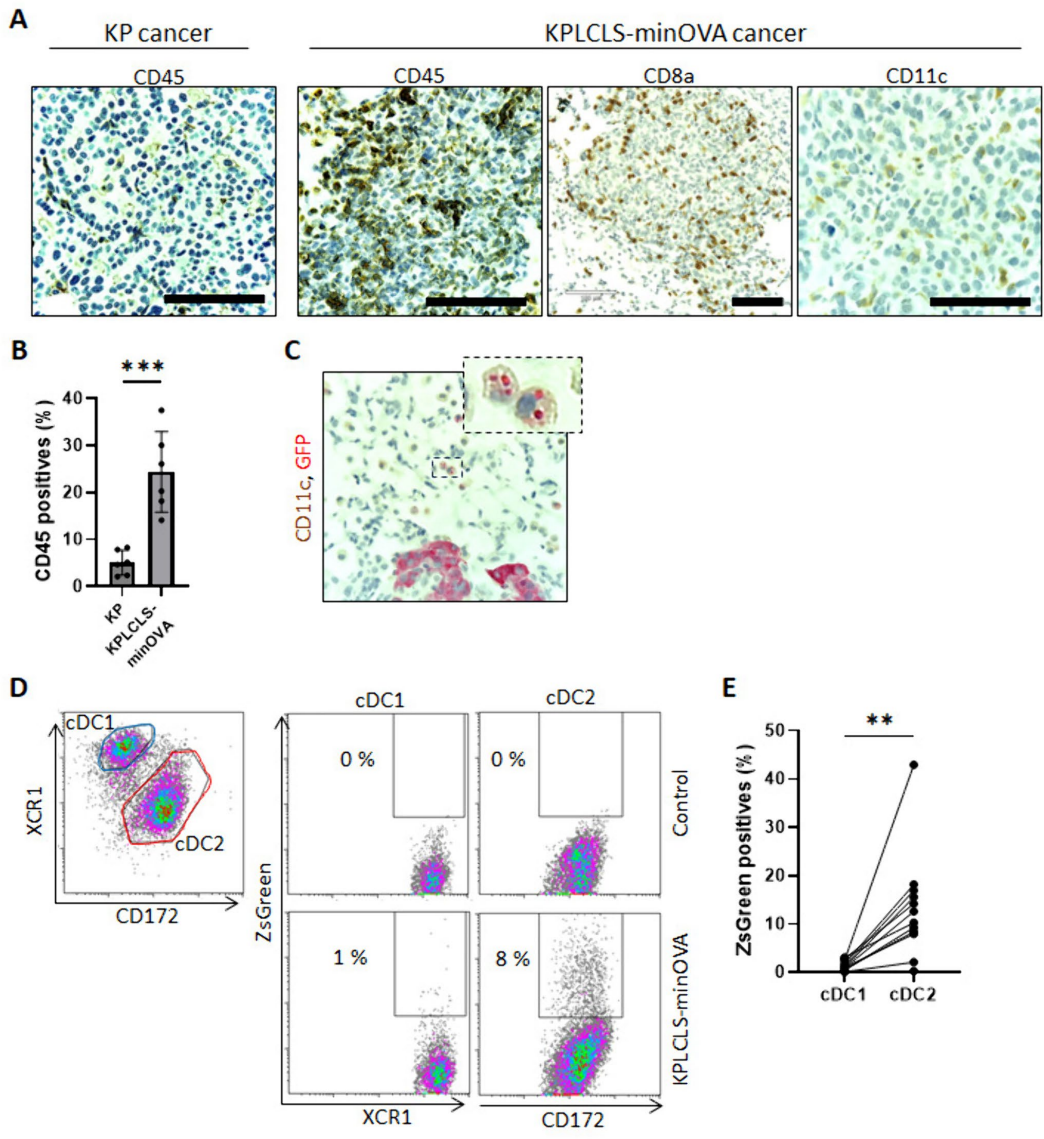
**Comparative analysis of immune cell infiltration in lung cancer models.** **A** Representative histological sections of lung cancer were stained with anti-CD45, anti-CD8a, or anti-CD11c antibodies. Lung cancer engrafted with KPLCLS-minOVA showed significant CD45 + immune cell infiltration compared to lung cancer induced by Tamoxifen injection (KP cancer). The tumor derived from KPLCLS-minOVA exhibits the infiltration of CD8a and CD11c immune cells. **B** Percentages of CD45 + were quantified using NIH ImageJ software. **C** KPLCLS-minOVA lung cancers were stained with anit-CD11c (brown) and anti-GFP (red) and showed intracellular GFP + signals. **D** Flow cytometry analysis demonstrates that CD172 + conventional dendritic cell type 2 (cDC2) cells engulf zsGreen + KPLCLS-minOVA cancer cells, whereas XCR1 + cDC1 cells show minimal uptake of cancer cells. (E) Percentages of zsGreen + were quantified. ** *P* < 0.002, ***P < 0.001 Scale bar: 100 µm

Immune cell composition in the tumor was analyzed using immunohistochemistry. Compared to the KP cancer cells, the KPLCLS-minOVA cell-derived tumors were found to have increased CD45^+^ immune cell infiltration (Fig. 4A & B). Immune cell infiltrates comprise CD8+ T cells and CD11c + cells (Fig. 4A). CD11c + cells are mononuclear cells without definite granules, suggesting dendritic cells in the lung, although some pulmonary macrophages express CD11c. Of interest, some CD11c^+^ cells contained intracellular GFP-positive granules, indicating engulfed tumor particles (Fig. 4C). To identify the subset of DCs that take up the tumor antigen, the tumor tissues were homogenized for flow cytometry. Cells were then gated for conventional DC populations using the surface markers, including CD45^+^CD11c^+^MHCII^hi^, and lineage negative (CD3, B220, NK1.1, TER119, CD64, F4/80). The cocktail excludes macrophages, T cells, B cells, and NK cells. The cDC population was further separated into lung cDC1 and cDC2 subsets using antibodies against XCR1 and CD172, as we previously reported [13]. ZsGreen positivity was observed mostly in the cDC2 subset, whereas cDC1 has minimal (Fig. 4D & E). These results indicate that tumor neoantigen is recognized by cDC2 cells, which may play a critical role in initiating antitumor immune responses.

## Discussion

In vivo animal models of cancer must strike a balance between tumor immunogenicity and reliability of growth. Highly mutated tumors may be more physiologic (particularly vis-à-vis lung cancer), but will be cleared by immunocompetent hosts at rates that render such models impractical for routine use. Similarly, minimally mutated tumors may exhibit consistent formation and growth, but lacking detectable neoantigens represent less realistic models and present little opportunity for the study of cancer immunology [4, 5]. Thus, an ideal tumor model should contain detectable neoantigens, but nevertheless demonstrate robust growth through the induction of tolerance, possibly even in the setting of immunotherapy, which has become the new standard of care in human disease. Furthermore, orthotopic and autochthonic models should also be preferred for the sake of human relevance, given the significant diversity in the character of immune responses arising from various anatomic niches. [14] Here, we present such a model for use in future studies of lung adenocarcinoma. Our tumor cells were generated from an initial autochthonic mouse model, then orthotopically delivered via intratracheal administration, and incorporated a carefully titrated neoantigen burden conducive to growth despite recognition by the immune system. Tumor cells express minOVA for the generation of OT-I and OT-II peptides for tracking CD8 + and CD4 + T cell responses, respectively, and additionally express ZsGreen for efficient recognition of tumor cells as well as phagocytes taking up tumor antigen. To our knowledge, possessing these features is highly relevant to human lung cancer and extremely valuable for studying tumor immune responses.

The KPLCLS-minOVA model offers significant advantages over existing models. It is derived from an orthotopic *Scgb1a*1-*Cre*^ERT^; *KP* mouse model, ensuring its origin from lung epithelial cells, which accurately reflects human lung adenocarcinoma. Additionally, the ZsGreen marker allows for tracking tumor antigen uptake within the tumor microenvironment. While, using a single neoantigen (minOVA) may not fully represent the diverse neoantigens in human lung cancers, this model effectively demonstrates increased immunogenicity and immune cell infiltration, providing a valuable tool for studying antitumor immune responses and developing immunotherapies. It is important to note that ZsGreen itself may potentially act as a neoantigen in mice, which could influence immune responses. While, this model should be broadly applicable to the study of lung adenocarcinoma and the immune responses engendered thereof, our preliminary studies to date have already highlighted an intriguing feature of the innate immune cells involved. In particular, we find that tumor antigen is selectively taken up by cDC2, rather than cDC1, which would be expected to skew the forthcoming adaptive immune response away from the generation of CD8 + T cells and Th1, and toward Th2, Th17, or Treg specification, thus impairing tumor clearance. [15] This phenomenon may account for our observation that these tumors, so reliably establish and progress upon delivery to the airway. Failure to generate a tumor infiltrative type 1 response has been recognized as a negative prognostic sign and correlates with tumor progression in humans. [16] Future studies will examine, whether cDC1 antigen uptake can be enhanced to promote tumor clearance or whether cDC2 can be inhibited toward the same end. Notably cDC2 are known to express significantly higher levels of PD-L1 than cDC1, [17] and this may relate to their ability under this model to establish a tolerogenic environment, permissive of tumor growth. While, our model is designed to facilitate analysis of T cell responses via OT-I and OT-II peptides, its greater novelty likely lies in its demonstrated ability to illustrate early contributions of the innate immune cells, such as DCs, which to date have received significantly less attention. Given that APCs are instrumental in determining T cell phenotype, our expectation is that future cancer therapies will arise from a better understanding of tumor antigen uptake and presentation by DCs.

## Supplementary Information

Below is the link to the electronic supplementary material.

Supplementary file1 (TIF 1022 KB)
