# Supplementary figures and images for: Tumor immunogenicity regulates host immune responses, and conventional dendritic cell type 2 uptakes the majority of tumor antigens in an orthotopic lung cancer model
Source: Cancer Immunol Immunother. 2024 Oct 3;73(12):237. doi: 10.1007/s00262-024-03828-w (PMC11447165; doi:10.1007/s00262-024-03828-w)

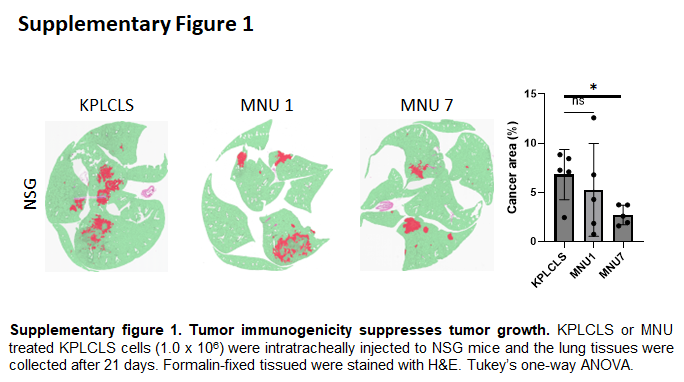

Supplement: Supplementary file 1 — Supplementary file1 (TIF 1022 KB) [file 262_2024_3828_MOESM1_ESM.tif]
